# HUWE1 Causes an Immune Imbalance in Immune Thrombocytopenic Purpura by Reducing the Number and Function of Treg Cells Through the Ubiquitination Degradation of Ets-1

**DOI:** 10.3389/fcell.2021.708562

**Published:** 2021-11-25

**Authors:** Jianqin Li, Yalin Xia, Xiaoru Fan, Xiaofang Wu, Feiyun Yang, Shaoyan Hu, Zhaoyue Wang

**Affiliations:** ^1^ Department of Hematology, The Children’s Hospital of Soochow University, Suzhou, China; ^2^ Department of Hematology, Jiangsu Institute of Hematology, The First Affiliated Hospital of Soochow University, Suzhou, China

**Keywords:** HUWE1, ETS-1, treg cells, immune thrombocytopenic purpura, CD4^+^

## Abstract

**Background:** Immune thrombocytopenic purpura (ITP) is an autoimmune bleeding disorder and the decreased number and immunosuppressive dysfunction of Treg cells are key promoters of ITP. However, their mechanisms in ITP development have not been fully clarified.

**Methods:** HUWE1 mRNA and protein levels in CD4^+^ T cells in peripheral blood from ITP patients were assessed by quantitative real-time PCR and Western blot. HUWE1 function in ITP was estimated using flow cytometry, enzyme-linked immunosorbent assay and immunosuppression assay. Besides, the HUWE1 mechanism in reducing the number and function of Treg cells in ITP was investigated by immunoprecipitation, cycloheximide-chase assay, ubiquitin experiment and immunofluorescence assay.

**Results:** HUWE1 expression was elevated in CD4^+^ T cells in peripheral blood from ITP patients and HUWE1 mRNA level was negatively correlated with platelet counts and Treg cell percentage. Moreover, the interference with HUWE1 increased the number of Treg cells and enhanced its immunosuppressive function, and the HUWE1 overexpression produced the opposite results. For the exploration of mechanism, HUWE1 interacted with E26 transformation-specific-1 (Ets-1) and this binding was dependent on the negative regulation of the phosphorylation level of Ets-1 (Thr38) and HUWE1 facilitated the ubiquitin degradation of Ets-1 protein to restrain Treg cell differentiation and weaken their immunosuppressive functions. The *in vivo* assay confirmed that the HUWE1 inhibitor alleviated ITP in mice.

**Conclusion:** HUWE1 induced the immune imbalance in ITP by decreasing the number and weakening the function of Treg cells through the ubiquitination degradation of Ets-1.

## 1 Introduction

Immune thrombocytopenic purpura (ITP) is an autoimmune disease characterized by platelet decrease and mucocutaneous bleeding (Blickstein, 2019; Wu et al., 2019). Traditionally, the imbalance of CD4^+^ T cell subsets exerts critical functions in the ITP pathogenesis (Wang et al., 2019; Kostic et al., 2020). Treg cells are a subset of CD4^+^ T cells and have pivotal immunosuppressive functions (Sakaguchi et al., 2010; Cheng et al., 2019). Previous studies demonstrate that the reduced number and dysfunction of Treg cells are key accelerating factors in the occurrence and development of autoimmune diseases, including ITP (Liu et al., 2007; Yu et al., 2008). Thus, elucidating the potential mechanism of ITP that causes the abnormal number and function of Treg cells is expected to ameliorate ITP.

HUWE1 is defined as the E3 ubiquitin ligase that contains the HECT domain and is interrelated to transcriptional regulation, cell apoptosis and immune signal transduction (Su et al., 2019). Due to the extensiveness and functional diversity of HUWE1 substrates, HUWE1 exerts momentous functions in human diseases (Kao et al., 2018). Previous studies corroborate that HUWE1 (also known as Mule) maintains the homeostasis of B lymphocytes through regulating the ATM-p53 axis, and shows its regulatory function in immune response (Hao et al., 2012); another study demonstrates that HUWE1 mediates the ubiquitination of its novel substrate KLF4 to participate in the proliferation and autoimmunity response of T cells (Hao et al., 2017), prompting that HUWE1 has a pivotal role in immune-related diseases. Interestingly, our research corroborated that HUWE1 was abnormally overexpressed in CD4^+^ T cells in peripheral blood from ITP patients, prompting that HUWE1 might be interrelated to the ITP progression. To verify this hypothesis, we further confirmed that the interference with HUWE1 elevated Treg cell number and enhanced its immunosuppressive function, while the HUWE1 overexpression reduced Treg cell number and weakened its immunosuppressive function. Based on these findings, we further investigated the regulatory mechanism of HUWE1 in Treg cell numbers and its function in ITP.

In the current research, we quantified the HUWE1 mRNA and protein levels in CD4^+^ T cells in peripheral blood from ITP patients and assessed the correlation between HUWE1 mRNA level and Treg cell percentage; and analyzed how HUWE1 regulated Treg cell number and its immunosuppressive function. Furthermore, we explored the HUWE1 mechanism in Treg cell number and function in ITP.

## 2 Materials and Methods

### 2.1 Clinical Samples

A total of 30 ITP patients were collected from the outpatient department of hematology of the Children’s Hospital of Soochow University between July 2018 and December 2019 and all the ITP patients met the clinical diagnosis criteria recommended by the 2011 American Society of Haematology guidelines (Neunert et al., 2011). Besides, a total of 30 healthy people in the same hospital physical examination center were selected as the control group. Peripheral blood samples were obtained with the consent of all patients and healthy people. This study was approved by the Ethics Committee of the Children’s Hospital of Soochow University (2020CS101).

### 2.2 CD4^+^ T Cell and Naive CD4^+^ T Cell Isolation

The 45 ml peripheral blood from ITP patients or healthy controls were gathered in a heparin tube and centrifuged at 1,200 g for 10 min. The supernatant containing white blood cells was mixed with phosphate-buffered saline (PBS, Thermo Fisher Scientific, MA, USA) and continued density gradient centrifugation to obtain peripheral blood mononuclear cells (PBMCs) and then were applied for the isolation of CD4^+^ T cells or Naive CD4^+^ T cells. Given the standard procedure of the reagent manufacturer, a human CD4^+^ T Cell Enrichment Kit (Stemcell, Beijing, China) was applied to isolate CD4^+^ T cells from PBMCs or a MagCellect Human Naive CD4^+^ T Cell Isolation Kit (Bio-Techne, MN, United States).

### 2.3 Cell Culture

Primary CD4^+^ T cells were placed in a complete RPMI 1640 (Procell, Wuhan, China) with 10% fetal bovine serum (FBS, Gibco, CA, United States), 50 U/ml penicillin (Gibco), 50 μg/ml streptomycin (Gibco) and 2 mM 1-glutamine (Thermo Fisher Scientific) and cultured in an incubator at 37°C and 5% CO_2_.

Jurkat T cells were from American Type Culture Collection (ATCC, United States). The cells were put in an RPMI-1640 medium (Procell) supplemented with 10% FBS (Gibco) in an incubator at 37°C with 5% CO_2_.

Primary Naïve CD4^+^ T cells were put in an RPMI 1640 (Procell) with the addition of 10% FBS (Gibco), 1% penicillin/streptomycin (Gibco) and 50 μM β-mercaptoethanol (Gibco) and cultured at 37°C and 5% CO_2_.

### 2.4 Different Treatment of Cells

TAK-733 is a selective MEK inhibitor (Jasek-Gajda et al., 2018) and GDC0994 is an inhibitor of ERK (Kirouac et al., 2017). Jurkat cells were treated with 1 μM TAK-733 (MedChemExpress, NJ, United States) or 1 μM GDC0994 (MedChemExpress) for 0, 1, 2, 4, 6 and 8 h.

Dasatinib is an Src family kinase inhibitor and Src can phosphorylate the Y283 of E26 transformation-specific-1 (Ets-1), activate Ets-1 and increase its stability (Lu et al., 2014; Appel et al., 2017). Jurkat cells were treated with 10 μM dasatinib (AbMole, Houston, TX, United States) for 0, 1, 2, 4, 6 and 8 h.

BI8622 is a specific inhibitor of HUWE1 (Peter et al., 2014). BI8622 with an IC_50_ of 3.1 μM was from MedChemExpress. Jurkat cells were treated with 1 μM BI8622.

### 2.5 Quantitative Real-Time PCR

Given the methods described in the previous literature (Wang et al., 2020), the qRT-PCR assay was conducted. Specifically, total RNA was isolated from harvested CD4^+^ T cells, Jurkat T cells and naive CD4^+^ T cells using TRIzol reagent (Solabio, Beijing, China). After quantifying the RNA concentration, the iScript cDNA synthesis kit (Bio-Rad, CA, United States) reverse-transcripted the RNA into cDNA. Then, SYBR Premix Ex Taq (TaKaRa, Shiga, Japan) was applied to run a real-time PCR on the 7500 real-time PCR system (Applied Biosystems, CA, United States) and analyzed the amplification products. After using the β-actin for standardization, a 2^−ΔΔCt^ method was applied to quantify the relative expression of different molecular. All the primer sequences are listed in Table 1.

**TABLE 1:** The sequences of all primers used in qRT-PCR.

Gene name	Primer sequence (5′-3′)
HUWE1	Forward: TGA​ATG​CTC​TGG​CTG​CAT​AC
	Reverse: CCCCAGGTTTAGGA TCAGATT
Foxp3	Forward: CCA​AGG​ATC​CTA​CCC​ACT​GCT​GG
	Reverse: CCCAGAGGTG CCTCCGCACTGC
CTLA-4	Forward: TGG​CCC​TGC​ACT​CTC​CTG​T
	Reverse: GGACCTCAGTGGCTTT GCCT
LAG3	Forward: CAA​TGG​CGA​CTT​TAC​CCT​TC
	Reverse: CCTCTGGGATGGGGT GTC
β-actin	Forward: CTC​CAT​CCT​GGC​CTC​GCT​GT
	Reverse: GCT​GTC​ACC​TTC​ACC​GTT​CC

### 2.6 Western Blot

Western blot experiment was performed given the previously described methods (Sun et al., 2019). CD4^+^ T cells, Jurkat T cells, naive CD4^+^ T cells and mouse spleen cells were harvested. The proteins were extracted with RIPA lysis buffer (Beyotime, Shanghai, China) and the protein concentrations were quantified using a bicinchoninic acid (BCA) Protein Assay Kit (TaKaRa). The same amount of protein samples were separated by SDS-polyacrylamide gel electrophoresis (SDS-PAGE, Thermo Fisher Scientific) and then transferred into polyvinylidene fluoride (PVDF) membranes (Thermo Fisher Scientific). The membranes were blocked with 5% skimmed milk and then incubated with anti-HUWE1 (ab70161, 1:2,000, Abcam), anti-FoxP3 (ab20034, 1:1,000, Abcam), anti-Ets-1 (ab220361, 1:1,000, Abcam), anti-p-Ets-1 (44-1107G, 1:1,000, ThermoFisher Scientific), anti-MEK1 (ab32576, 1:10,000, Abcam) and anti-GAPDH (ab8245, 1:500, Abcam) overnight at 4°C. The membranes were washed with TBST and incubated with the secondary antibody (ab205718, 1:2,000, Abcam) for 1 h at room temperature. The enhanced chemiluminescence reagents (Thermo Fisher Scientific) and the Versadoc MP 4000 imaging system (Bio-Rad) were performed to visualize protein bands.

### 2.7 Detection of Platelet Counts

Peripheral blood from each ITP patient or ITP mouse was collected into a sample collection tube fitted with EDTA. After that, an automated cell analyzer (Beckman Coulter, DxH 800, FL, United States) was applied to conduct the platelet counts.

### 2.8 Flow Cytometry

The cell density was adjusted to 1 × 10^6^/100 μl/flow tubes, and 20 μl CD4 PE-Cy5/CD25 PE cocktail antibody (Thermo Fisher Scientific) was added to each tube. After thoroughly mixing, the cells were incubated at room temperature in dark for 20 min. After the cells were washed, the supernatant was discarded after centrifugation at 250 ×g for 5 min. Then, 1 ml of fixation/permeabilization working solution (BD Pharmingen, NJ, United States) was added and incubated in dark at room temperature for 20 min, followed by centrifugation at 250 ×g for 5 min. Then the supernatant was discarded. The cells were washed with 1 ml permeabilization buffer (Thermo Fisher Scientific), centrifuge at 250 ×g for 5 min and further discard the supernatant. Subsequently, the cells were resuspended with 100 μl permeabilization buffer (Thermo Fisher Scientific) and 5 μl Alexa Fluor® 488 Foxp3 antibody (Thermo Fisher Scientific) or 5 μl Alexa Fluor® 488 IgG (Abcam) was added to each tube and incubated at room temperature in dark for 30 min. Ultimately, the cells were washed twice with cell staining buffer and were resuspended with 0.5 ml of cell staining buffer for detection on flow cytometry BD FACSCanto II (BD, Franklin Lakes, NJ, United States).

### 2.9 Cell Transfection

PlentiLox 3.7 vector was purchased from Axibio (Hunan, China). Human HUWE1, Ets-1 and MEK-1 containing full-length open reading frame and termination codon was inserted into the upper stream of GFP in PlentiLox 3.7 vector and was started by CMV Promoter and named Lenti-HUWE1, Lenti-Ets-1 and Lenti-MEK1. The HUWE1 shRNA sequence was: 5′-AAU​UGC​UAU​GUC​UCU​GGG​ACA-3′.

Before conducting lentivirus transfection experiments, we determined the multiplicity of infection (MOI) by pre-experiment and selected the MOI value of 50 to start the follow-up experiment. Based on the standard procedures of manufacturers, we conducted all lentivirus transfections.

### 2.10 Enzyme-Linked Immunosorbent Assay

Given the reagent manufacturer’s instructions, the concentration of IL-10 in cell culture supernatants of CD4^+^ T cells in the peripheral blood from ITP patients or healthy controls was quantified using an IL-10 ELISA kit (Mlbio, Shanghai, China).

### 2.11 Immunosuppression Assay

Based on the previously described methods with minor modifications (Zhang et al., 2015), the immunosuppression experiments were performed. HUWE1 shRNA was transfected into Treg cells and then co-cultured with effector cell T at a ratio of 1:4. Then, the immunosuppression was carried out using a Treg Suppression Inspector Kit (Miltenyi Biotec, Germany) as the reagent manufacturer’s instructions. The inhibitory effect of Treg cells on the proliferation of effector T cells was assessed using flow cytometry (BD).

### 2.12 Immunoprecipitation

The binding of HUWE1 to Ets-1 in different treated CD4^+^ T cells and Jurkat T cells was analyzed by immunoprecipitation (IP) assay. After the cells were lysed in RIPA buffer, the cell lysates were incubated with anti-Ets-1 (Abcam), followed by incubation with 100 μl protein A magnetic beads (Thermo Fisher Scientific) at room temperature for 1 h. The beads were collected after centrifugation and washed three times with a lysis buffer. The eluted immune complexes were tested using Western blot.

### 2.13 Cycloheximide-Chase Assay

Cycloheximide (CHX) is a commonly used protein synthesis inhibitor (Nikitin et al., 2019). To analyze the influence of HUWE1 on the Ets-1 stability, the CHX-chase assay was conducted. Lenti-HUWE1 and the corresponding control group Lenti were transfected into Jurkat cells for 48 h and then the cells were treated with 50 mmol/l CHX (MedChemExpress) for 0, 1, 2, or 4 h. The Ets-1 protein level was quantified by Western blot.

### 2.14 Ubiquitin Experiment

Ub-HA and/or Lenti-HUWE1, Ub-HA and/or Lenti-HUWE1 C4341A were transfected into Jurkat T cells for 48 h and then the endogenous Ets-1 ubiquitination was tested. HUWE1 C4341A refers to the change of the base aca tgt to ac*c* *gc*t by site-directed mutagenesis to introduce the C to A mutation of amino acid 4341 in HUWE1 (also named Mule), which removes the E3 ligase activity of HUWE1 (Hao et al., 2017).

To verify the HUWE1 regulation on Ets-1 ubiquitination, Lenti-HUWE1 and its control group Lenti were transfected into Jurkat cells for 48 h, and 10 μM protease inhibitor MG132 (MedChemExpress) was added to continue processing the cells for 4 h before harvesting the cell samples. Ultimately, the Ets-1 protein level was tested using Western blot.

### 2.15 Immunofluorescence Assay

After lenti-HUWE1 was transfected into Jurkat T cells for 48 h, the Ets-1 expression and its nucleation were assessed by immunofluorescence analysis. Specifically, the Jurkat T cells were harvested and washed twice with PBS. Then the cells were fixed with 4% paraformaldehyde (Thermo Fisher Scientific) for 15 min and permeabilized with 0.2% Triton X-100 (Thermo Fisher Scientific) for 5 min. After that, the cells were blocked with 5% bovine serum albumin (BSA, Gibco) for 30 min at room temperature. The cell samples were incubated with the primary antibody (anti-Ets-1, ab186844, Abcam) overnight at 4°C; the cells were then incubated with the secondary antibody (ab205718, Abcam) for 30 min at room temperature. Besides, the nucleus was stained with 4′,6-diamidino-2-phenylindole (DAPI, Beyotime). All the images were taken and saved by an immunofluorescence microscope (Olympus, Tokyo, Japan).

### 2.16 Establishment of a Mouse ITP Model

Referring to the previously reported methods (Zhao et al., 2019), we constructed the mouse model of ITP. Sixteen male C57BL/6J mice (6–8 weeks old) were divided into ITP and ITP + BI8622 groups. Eight mice were randomly assigned to each group. The mice (platelet count 8.5–12.5×10^11^/l) were intravenously injected with an anti-platelet monoclonal antibody (monoclonal antibody, rat anti-mouse CD41, clone MWReg30; BD Biosciences). The initial intravenous dose was 0.3 mg/kg mouse body weight and the subsequent dose was 0.1 mg/kg every 36 h. Moreover, to analyze the effect of HUWE1 inhibitor on ITP mice, HUWE1 specific inhibitor BI8622 was intraperitoneally injected during the establishment of the mouse ITP model (the inhibitor was given at the same time of the first antibody injection, 3 times a week, at the injection dose of 0.1 mg/kg). On the 15th day of ITP model construction, all mice were sacrificed and their spleen tissues were isolated for the following studies. All animal protocols were approved by the Animal Protection and Use Committee of the Ethics Committee of Soochow University and were conducted in accordance with the Animal Protection and Use Guidelines of the Ethics Committee of Soochow University (ECSU-2019000222).

### 2.17 Hematoxylin-Eosin Staining

After the euthanasia of ITP mice, the spleen tissues of mice were isolated. The tissues were then fixed with 4% paraformaldehyde (Thermo Fisher Scientific) and made into 5 μm thick slices. Immediately after, a Hematoxylin and Eosin Staining Kit (Beyotime) was conducted to stain the slices. The pathological changes of spleen tissues in mice were observed by the randomly selected three fields under a microscope (Olympus).

### 2.18 Statistical Analysis

All data are assessed using SPSS v.25 unless otherwise stated. All data are expressed as mean ± standard error of the mean (SEM). When the differences between the two groups were compared, a two-tailed Unpaired Student’s *t*-test was performed; when the differences among more than two groups were compared, one-way analysis of variance (ANOVA) followed by the Tukey’s post-hoc test was conducted. If *p*-value was less than 0.05, the difference is considered to be significant.

## 3 Results

### 3.1 HUWE1 Is Highly Expressed in CD4^+^ T Cells in Peripheral Blood From ITP Patients

**FIGURE 1:**
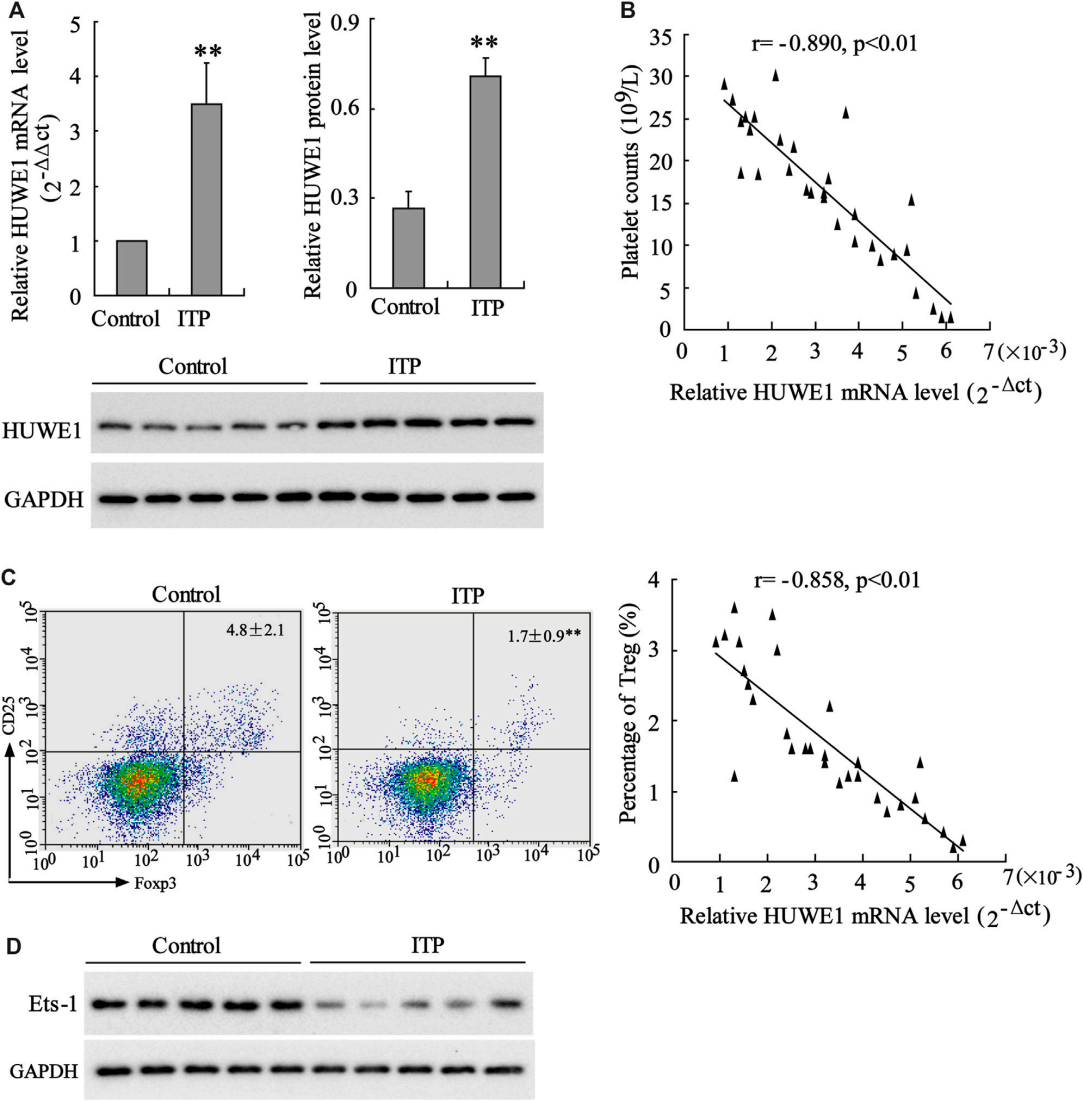
Expression of HUWE1 in CD4^+^ T cells in peripheral blood from immune thrombocytopenic purpura patients. **(A)** Quantitative real-time PCR (qRT-PCR) and Western blot assays were performed to detect the mRNA and protein levels of HUWE1 in CD4^+^ T cells in the peripheral blood from healthy controls and immune thrombocytopenic purpura (ITP) patients; and the quantitative analysis of HUWE1 protein level (mean ± SEM, *n* = 5). **(B)** Correlation analysis of the mRNA level of HUWE1 and platelet counts in CD4^+^ T cells in the peripheral blood from ITP patients (*r* = −0.890, *p* < 0.01), *n* = 30. **(C)** Flow cytometry was applied to analyze the percentage of Treg cells in CD4^+^ T cells in peripheral blood from ITP patients and correlation analysis of the mRNA level of HUWE1 and the percentage of Treg cells in CD4^+^ T cells in peripheral blood from ITP patients (*r* = −0.858, *p* < 0.01), *n* = 30. **(D)** Western blot was performed to assess the Ets-1 protein level in the CD4^+^ T cells in the peripheral blood from ITP patients. The experiment was repeated three times. GAPDH is applied as the loading control. **p < 0.01 vs. control. ITP, immune thrombocytopenic purpura.

HUWE1 mRNA and protein levels were assessed in CD4^+^ T cells in the peripheral blood from healthy controls and ITP patients. The results revealed that HUWE1 expression in CD4^+^ T cells in the peripheral blood from ITP patients was higher than that from healthy controls (Figure 1A), and the quantitative analysis of HUWE1 protein level was displayed in Figure 1A. In the CD4^+^ T cells in the peripheral blood from ITP patients, the mRNA level of HUWE1 was negatively correlated with platelet counts (*n* = 30) (Figure 1B). Furthermore, the Treg cell percentage was decreased in CD4^+^ T cells in peripheral blood from ITP patients, and the mRNA level of HUWE1 was negatively correlated with the Treg cell percentage (*n* = 30) (Figure 1C). In the CD4^+^ T cells in peripheral blood from ITP patients, the Ets-1 protein level was decreased (Figure 1D). These results corroborated that HUWE1 expression was elevated in the CD4^+^ T cells in peripheral blood from ITP patients and was negatively correlated with platelet counts and Treg cell percentage.

### 3.2 Regulation of HUWE1 Knockout on Treg Cell Percentage, Foxp3 Expression, IL-10 Production and Treg Cell Immunosuppressive Function

**FIGURE 2:**
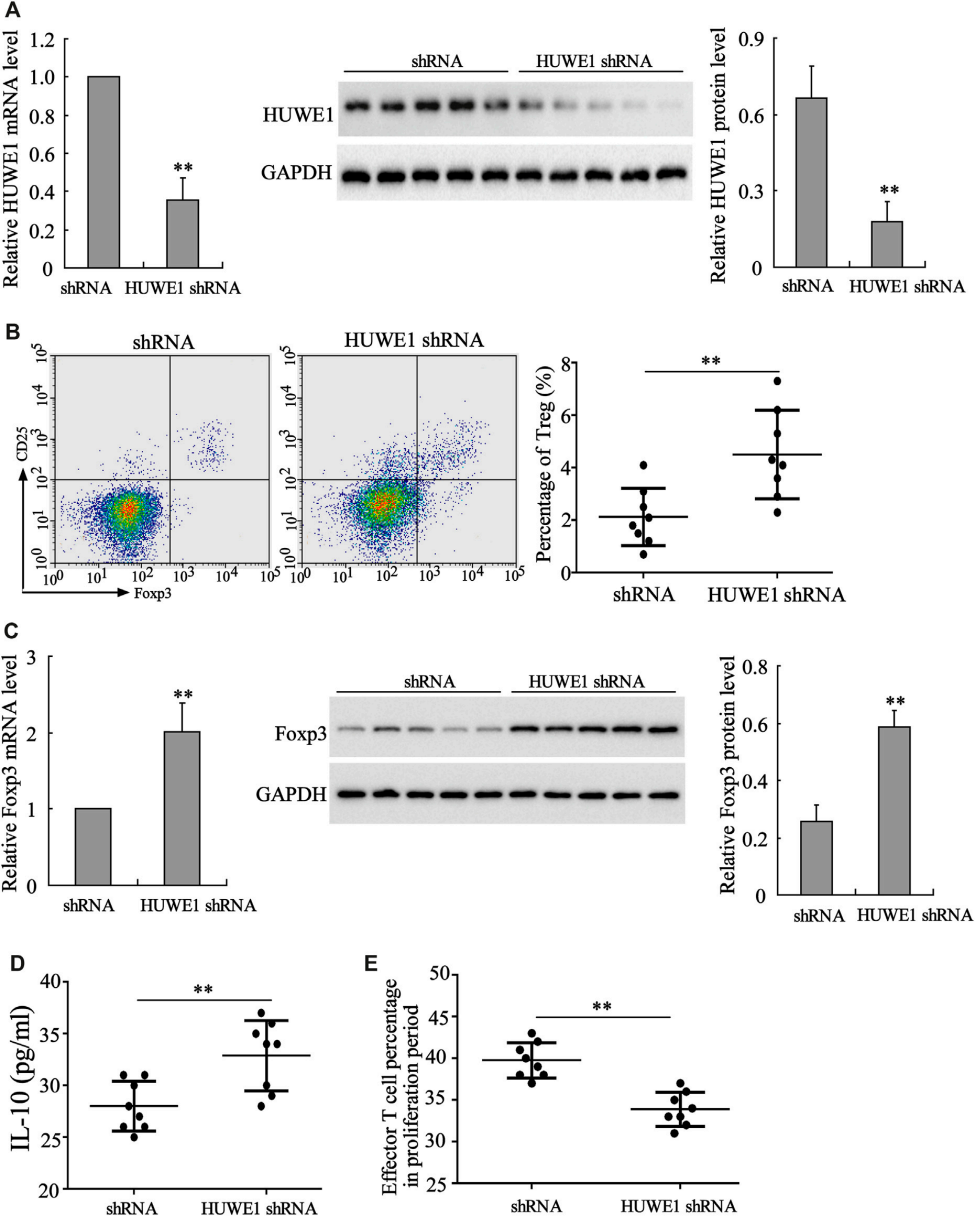
Influence of HUWE1 on Treg cell percentage, Foxp3 expression, IL-10 production and Treg cell immunosuppressive function. CD4^+^ T cells in peripheral blood from ITP patients were transfected with HUWE1 shRNA for 96 h. **(A)** Analysis of the HUWE1 mRNA and protein levels using qRT-PCR and Western blot; and the quantitative analysis of HUWE1 protein level (mean ± SEM, *n* = 5). **(B)** Flow cytometry was conducted to analyze the percentage of Treg cells in CD4^+^ T cells (mean ± SEM, *n* = 8). **(C)** qRT-PCR and Western blot were performed to quantify the mRNA and protein levels of Foxp3 in CD4^+^ T cells; and the quantitative analysis of Foxp3 protein level (mean ± SEM, *n* = 5). **(D)** Enzyme-linked immunosorbent assay (ELISA) was applied to detect the concentration of IL-10 in CD4^+^ T cell culture supernatant (mean ± SEM, *n* = 8). **(E)** Treg cells from ITP patients were transfected with HUWE1-interfering lentivirus (HUWE1 shRNA) and then cultured with effector T cells in a ratio of 1:4. Immunosuppression assay was performed to analyze the inhibitory effect of Treg cells on the proliferation of effector T cells (mean ± SEM, *n* = 8). **p < 0.01 vs. shRNA. The experiment was repeated three times.

CD4^+^ T cells in peripheral blood from ITP patients were transfected with HUWE1 shRNA. As displayed in Figure 2A, the HUWE1 mRNA and protein levels were decreased after the HUWE1 knockdown. Besides, the flow cytometry results confirmed that CD4^+^ T cells in peripheral blood from ITP patients transfected with HUWE1 shRNA elevated Treg cell percentage (Figure 2B). Foxp3 exerts a pivotal role in regulating the development and function of Treg cells (Nie et al., 2015). qRT-PCR and Western blot results authenticated that the interference with HUWE1 elevated the mRNA and protein levels of Foxp3 in CD4^+^ T cells (Figure 2C), and the quantitative analysis of Foxp3 protein level was displayed in Figure 2C. Besides, the interference with HUWE1 increased the IL-10 concentration in the CD4^+^ T cell culture supernatant (Figure 2D). Furthermore, the immunosuppression assay clarified that the HUWE1 knockdown accelerated the inhibitory effect of Treg cells on the proliferation of effector T cells (Figure 2E). Meanwhile, CD4^+^ T cells in peripheral blood from ITP patients transfected with HUWE1 shRNA elevated the CTLA-4 and LAG3 expressions (Supplementary Figure S1A). In conclusion, the HUWE1 knockdown elevated Treg cell percentage, Foxp3 expression and IL-10 production and enhanced the inhibitory effect of Treg cells on the proliferation of effector T cells.

### 3.3 Influence of HUWE1 Overexpression on Treg Cell Percentage, Foxp3 Expression, IL-10 Production and Treg Cell Immunosuppressive Function

**FIGURE 3:**
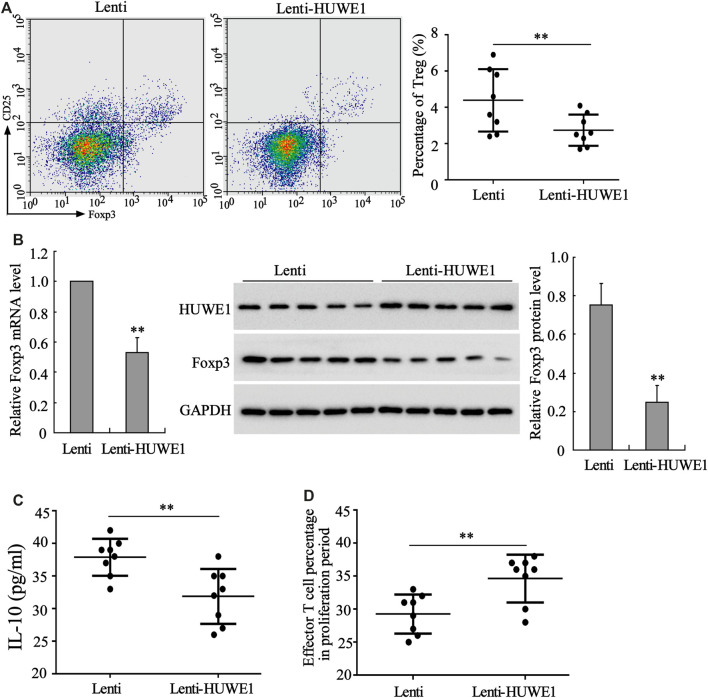
Effect of HUWE1 overexpression on Treg cell percentage, Foxp3 expression, IL-10 production and Treg cell immunosuppressive function. CD4^+^ T cells in peripheral blood from healthy controls were transfected with Lenti-HUWE1 for 96 h. **(A)** Flow cytometry was performed to detect the percentage of Treg cells in CD4^+^ T cells (mean ± SEM, *n* = 8). **(B)** qRT-PCR was conducted to measure the mRNA level of Foxp3 in CD4^+^ T cells and Western blot was carried out to measure the protein levels of Foxp3 and HUWE1 in CD4^+^ T cells; and the quantitative analysis of Foxp3 protein level (mean ± SEM, *n* = 5). **(C)** ELISA was applied to detect the concentration of IL-10 in the CD4^+^ T cell culture supernatant (mean ± SEM, *n* = 8). **(D)** An immunosuppression experiment was conducted to assess the inhibitory effect of Treg cells on the proliferation of effector T cells (mean ± SEM, *n* = 8). **p < 0.01 vs. Lenti. The experiment was repeated three times.

CD4^+^ T cells in peripheral blood from healthy controls were transfected with Lenti-HUWE1. Flow cytometry analysis expounded that CD4^+^ T cells in peripheral blood from ITP patients transfected with Lenti-HUWE1 decreased Treg cell percentage (Figure 3A). As exhibited in Figure 3B, the HUWE1 overexpression elevated the HUWE1 protein level and lessened the mRNA and protein levels of Foxp3 in CD4^+^ T cells; and the quantitative analysis of Foxp3 protein level was displayed in Figure 3B. Subsequently, we authenticated that the HUWE1 overexpression decreased the IL-10 concentration in the CD4^+^ T cell culture supernatant (Figure 3C). Meanwhile, the HUWE1 overexpression weakened the inhibitory effect of Treg cells on the proliferation of effector T cells (Figure 3D). Furthermore, CD4^+^ T cells in peripheral blood from healthy controls transfected with Lenti-HUWE1 decreased the CTLA-4 and LAG3 expressions (Supplementary Figure S1B). In general, the HUWE1 overexpression lessened Treg cell percentage, Foxp3 expression and IL-10 production and restrained the inhibitory effect of Treg cells on the proliferation of effector T cells.

### 3.4 HUWE1 Interacts With Ets-1

**FIGURE 4:**
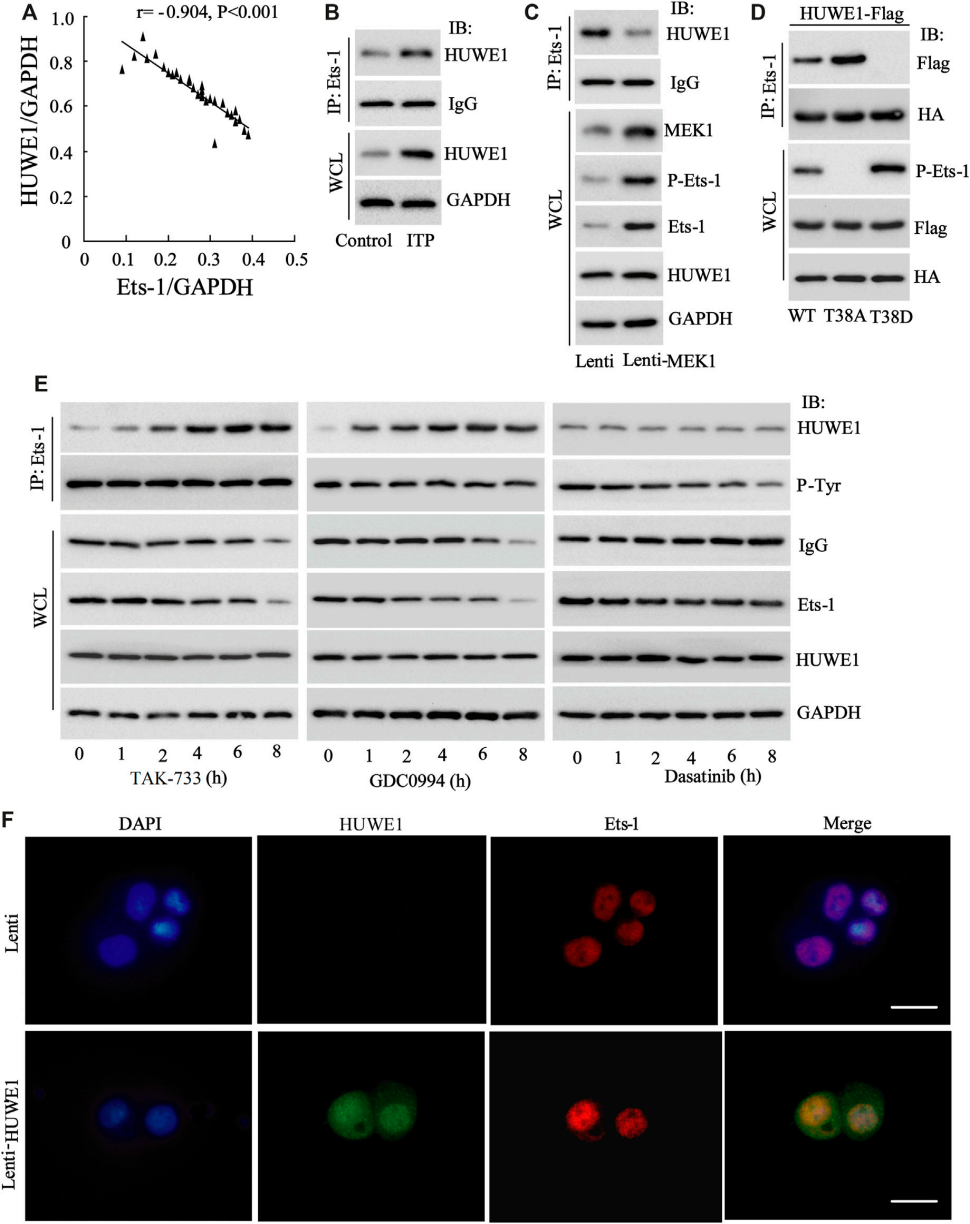
The interaction between HUWE1 and E26 transformation-specific-1 (Ets-1). **(A)** Correlation analysis of the protein level of HUWE1 and the protein level of Ets-1 in the CD4^+^ T cells in the peripheral blood from ITP patients (*r* = −0.904, *p* < 0.001), *n* = 30. **(B)** Immunoprecipitation (IP) was performed to analyze the binding of HUWE1 to Ets-1 in CD4^+^ T cells in peripheral blood from healthy controls or ITP patients. **(C)** Lenti-MEK1 (a constitutively activated MEK1) was transfected into Jurkat T cells and the transfection efficiency was verified by Western blot. IP was conducted to assess the binding of HUWE1 to Ets-1 and a Western bolt assay was applied to detect the protein levels of p-Ets-1 (T38 sites), Ets-1 and HUWE1. **(D)** 293A cells were transfected with WT (Ets-1 wild-type plasmid) and HUWE1-Flag, T38A (point mutant plasmid phosphorylation inactivation at T38 of Ets-1: mutates T to A) and HUWE1-Flag, or T38D (point mutant plasmid phosphorylation activation at T38 of Ets-1: mutates T to D) and HUWE1-Flag for 24 h, HA for IP followed by Flag for immunoblotting (IB) or Flag for IP followed by HA for IB. The analysis of the binding of HUWE1 to Ets-1 WT, to Ets-1 T38A or Ets-1 T38D. HA: Both the WT plasmid and Mut plasmid of Ets-1 had HA tags. **(E)** Jurkat T cells were treated with 1 μM MEK inhibitor TAK-733, 1 μM ERK inhibitor GDC0994, and 10 μM the Src family kinase inhibitor dasatinib for 0, 1, 2, 4, 6 and 8 h, respectively. The analysis of the binding of HUWE1 to Ets-1. **(F)** Immunofluorescence confirmed the overexpression of HUWE1 and its co-localization with ETS-1 in the nucleus (scale bar: 10 μm). IP, immunoprecipitation; IB, immunoblotting; WCL, whole cell lysate. The experiment was repeated three times.

Next, we tried to probe into the specific mechanism by which HUWE1 functioned in the immunosuppression of ITP. Studies indicate that Ets-1 is a key molecule that mediates immune homeostasis (Polansky et al., 2010; Moisan et al., 2007). The HUWE1 protein level was negatively correlated with the Ets-1 protein level (*n* = 30) (Figure 4A), implying that there might be a regulatory relationship between HUWE1 and Ets-1. To further verify this hypothesis, we conducted the IP assay and corroborated that the binding of HUWE1 to Ets-1 might be enhanced in CD4^+^ T cells in peripheral blood from ITP patients (Figure 4B). Previous studies demonstrate that the activation of the MEK/ERK signaling pathway phosphorylates the T38 sites of Ets-1 and activates Ets-1, thereby elevating the Ets-1 expression (Li et al., 2019). In the current study, the Lenti-MEK1 (a constitutively activated MEK1) was transfected into Jurkat T cells and the transfection efficiency was verified using Western blot (Figure 4C). After the Lenti-MEK1was transfected into Jurkat T cells, p-Ets-1 (T38 sites) and Ets-1 expressions were elevated, while the binding of HUWE1 to Ets-1 was weakened and HUWE1 expression had no remarkable changes (Figure 4C). As shown in Figure 4D, WT (Ets-1 wild-type plasmid) and T38A (point mutant plasmid phosphorylation inactivation at T38 of Ets-1: mutates T to A) bound to HUWE1, while T38D (point mutant plasmid phosphorylation activation at T38 of Ets-1: mutates T to D) could not bind to HUWE1. Furthermore, Jurkat T cells were treated with the MEK inhibitor TAK-733, ERK inhibitor GDC0994 and the Src family kinase inhibitor dasatinib. As exhibited in Figure 4E, after the treatment with TAK-733 and GDC0994, the binding of HUWE1 to Ets-1 was facilitated; after the treatment with dasatinib, the binding of HUWE1 to Ets-1 had no prominent changes. Furthermore, the immunofluorescence results demonstrated that HUWE1 was overexpressed and co-localized with Ets-1 in the nucleus (Figure 4F). In general, HUWE1 bound to Ets-1 and the binding was dependent on the phosphorylation level of Ets-1.

### 3.5 HUWE1 Facilitates the Ubiquitination and Degradation of Ets-1 Protein

**FIGURE 5:**
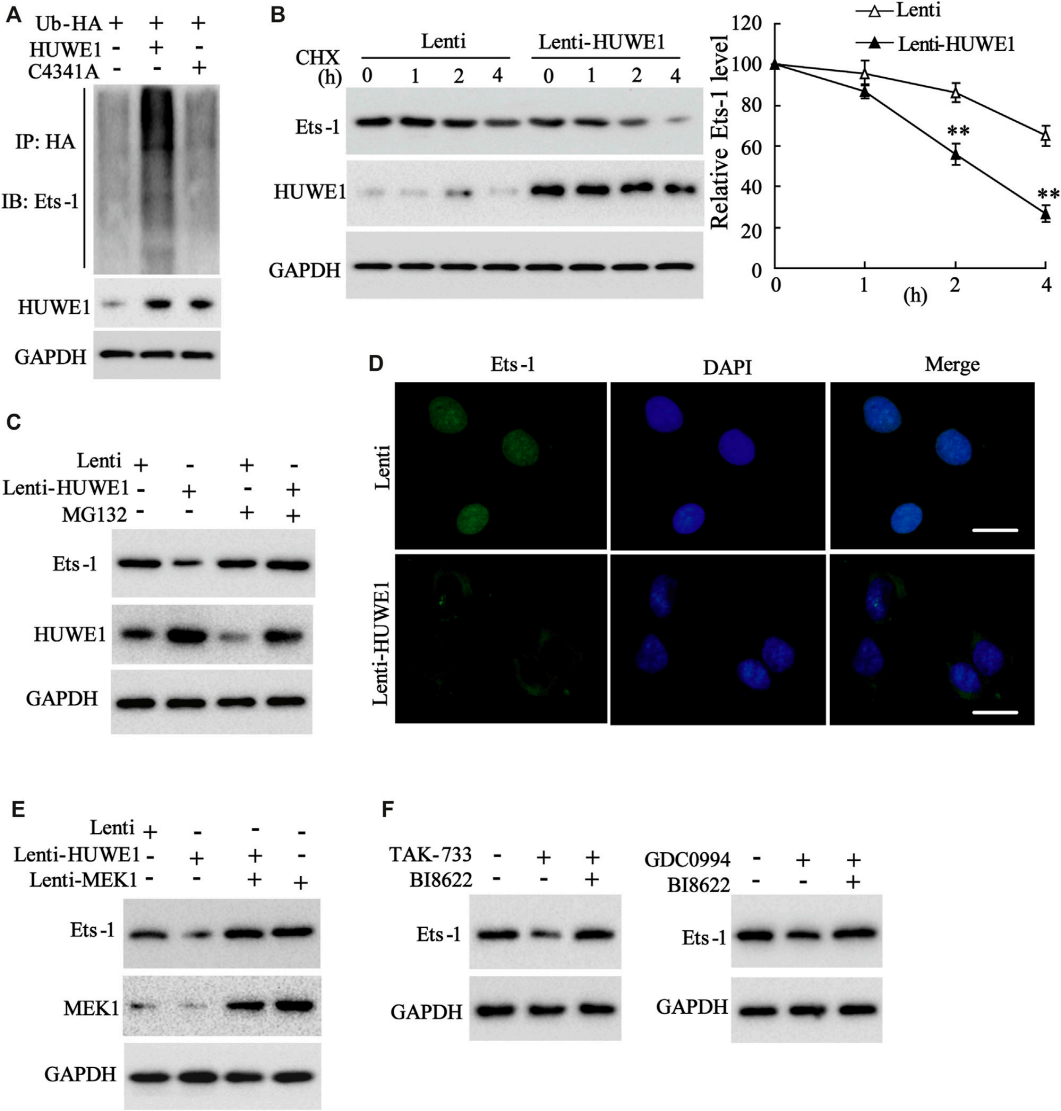
Influence of HUWE1 on the ubiquitination and degradation of Ets-1 protein. **(A)** Jurkat T cells were transfected with Hb-HA and Lenti-HUWE1 or Lenti-HUWE1C4341A, respectively. Ubiquitin experiment was conducted to assess the ubiquitination of Ets-1. **(B)** Jurkat T cells were transfected with Lenti-HUWE1 for 48 h and then were treated with 50 mmol/L CHX for 0, 1, 2 and 4 h. Western blot was performed to measure the protein levels of Ets-1 and HUWE1. **(C)** Jurkat T cells were transfected with Lenti-HUWE1 for 48 h and then were treated with 10 μm MG132 4 h before the sample harvesting. Western blot was applied to detect the protein level of Ets-1 and HUWE1. **(D)** Jurkat T cells were transfected with Lenti-HUWE1 for 48 h. An immunofluorescence assay was conducted to analyze the expression of Ets-1 and the nuclear export of Ets-1 (scale bar: 10 μm). **(E)** Jurkat T cells were transfected with Lenti-HUWE1 and/or Lenti-MEK1. Western blot was applied to detect the protein levels of Ets-1 and MEK1. **(F)** Jurkat T cells were treated with TAK-733 (1 μM) and/or HUWE1 specific inhibitor BI8622 (1 μM), GDC0994 (1 μM) and/or BI8622 (1 μM). Western blot was performed to detect the protein level of Ets-1. **p < 0.01 vs. Lenti. The experiment was repeated three times. CHX: cycloheximide.

Subsequently, we further elucidated how HUWE1 regulated the Ets-1 expression. C4341A refers to site-directed mutagenesis that converts cysteine at amino acid 4341 of HUWE1 into alanine, thus eliminating the E3 ligase activity of HUWE1 (Hao et al., 2017). Next, Jurkat T cells were transfected with Hb-HA and Lenti-HUWE1 or Lenti-HUWE1C4341A. As displayed in Figure 5A, the HUWE1 overexpression facilitated the Ets-1 ubiquitination, but the HUWE1 C4341A overexpression did not. CHX-chase assay illustrated that the HUWE1 overexpression accelerated the Ets-1 protein degradation (Figure 5B), and the transfection efficiency of Lenti-HUWE1 was verified using Western blot (Figure 5B). MG132 is a widely used proteasome inhibitor (Sun et al., 2018). As exhibited in Figure 5C, the HUWE1 overexpression lessened the Ets-1 expression, while the MG132 treatment elevated the Ets-1 expression, hinting that HUWE1 modified Ets-1 by ubiquitination, resulting in the degradation of Ets-1 by the proteasome. Besides, the HUWE1 overexpression decreased Ets-1 expression and facilitated the nuclear export of Ets-1 (Figure 5D). Subsequently, Jurkat T cells were transfected with Lenti-HUWE1 and/or Lenti-MEK1. The Western blot analysis illustrated that the transfection of Lenti-HUWE1 alone induced a decrease in the Ets-1 protein level, while the simultaneous transfection of Lenti-MEK1 could not (Figure 5E); and the transfection efficiency of Lenti-MEK1 was confirmed by Western blot (Figure 5E). Furthermore, Jurkat T cells were treated with TAK-733 and/or HUWE1 specific inhibitor BI8622, GDC0994 and/or BI8622. The results expounded that both TAK-733 and GDC0994 lessened the Ets-1 protein level, and this trend was reversed by the BI8622 addition (Figure 5F). In brief, the HUWE1 overexpression accelerated the ubiquitination and degradation of the Ets-1 protein.

### 3.6 HUWE1 Restrains the Differentiation and Function of Treg Cells by Lessening Ets-1

**FIGURE 6:**
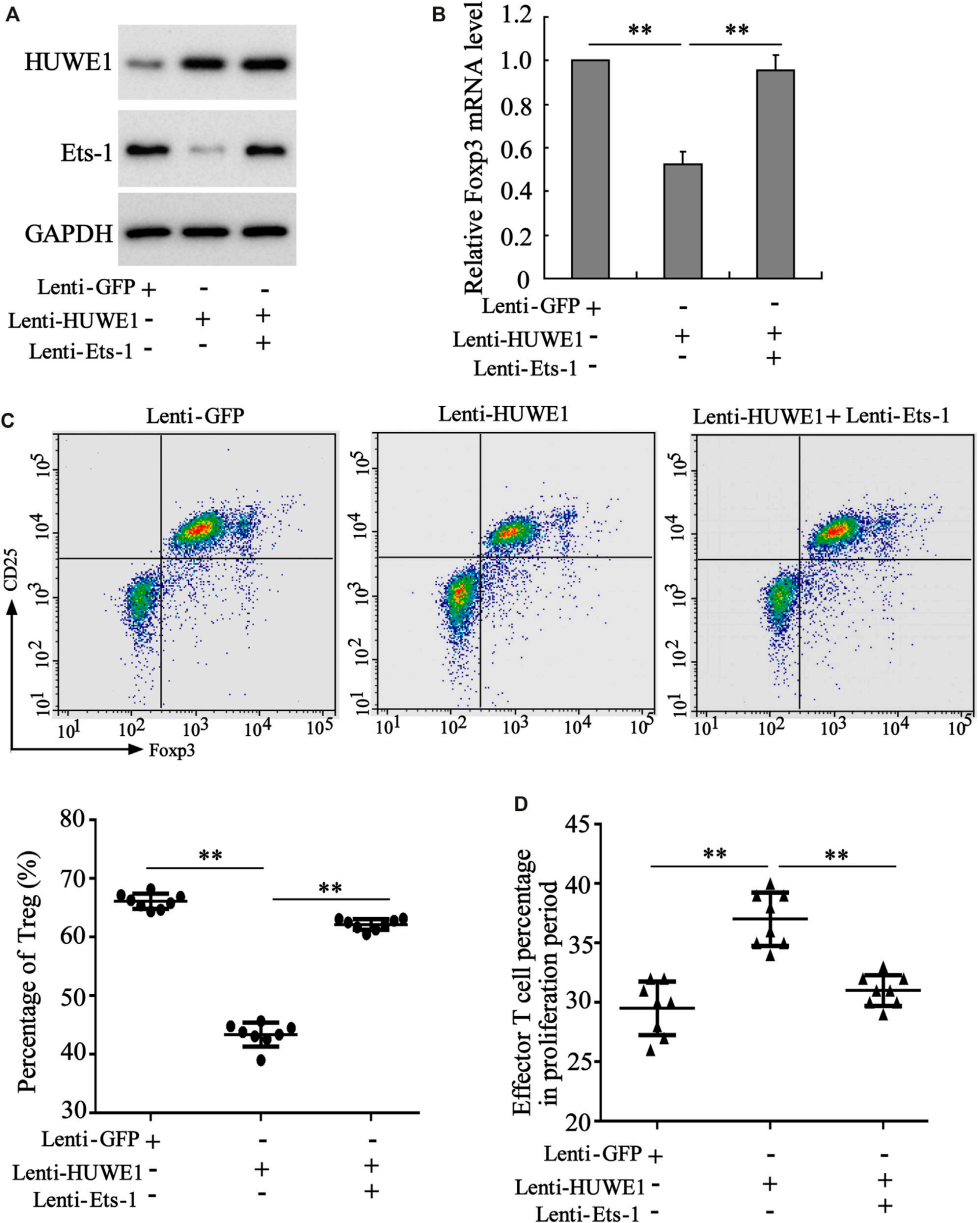
Regulation of HUWE1 on the differentiation and function of Treg cells through Ets-1. Naive CD4^+^ T cells were isolated from the peripheral blood of healthy controls and transfected with HUWE1 lentivirus and/or Ets-1 lentivirus for 48 h, and were cultured in Treg polarization conditions (cells in the presence of plate-bound anti-CD3, solid anti-CD28, TGF β (5 ng/ml), and IL-2 (50 IU/ml) for 72 h. **(A)** Western blot was applied to measure the protein levels of HUWE1 and Ets-1. **(B)** qRT-PCR was performed to quantify the mRNA level of Foxp3 (mean ± SEM, *n* = 3). **(C)** Flow cytometry was conducted to assess the percentage of Treg cells in Naive CD4^+^ T cells (mean ± SEM, *n* = 8). **(D)** Treg cells from the peripheral blood of healthy controls were transfected with HUWE1 lentivirus and/or Ets-1 lentivirus for 48 h and then cultured with effector T cells in a ratio of 1:4. Immunosuppression assay was performed to analyze the inhibitory effect of Treg cells on the proliferation of effector T cells (mean ± SEM, n = 8). **p < 0.01 vs. Lenti or Lenti-HUWE1. The experiment was repeated three times.

To determine whether Ets-1 mediated the differentiation and function of Treg cells induced by the HUWE1 overexpression, CD4^+^ T cells were isolated from the peripheral blood of healthy controls and transfected with Lenti-HUWE1 and/or Lenti-Ets-1 and were cultured in Treg polarization conditions. Western blot analysis demonstrated that the HUWE1 overexpression elevated the HUWE1 protein level and decreased Ets-1, and the Ets-1 overexpression increased the Ets-1 protein level (Figure 6A). Previous studies confirm that Foxp3 regulates the differentiation and function of Treg cells (Ziegler and Buckner, 2009; Ono, 2020). As displayed in Figure 6B, the HUWE1 overexpression decreased the mRNA level of Foxp3, while this trend was reversed after the transfection of Lenti-Ets-1. Based on this finding, we further conducted flow cytometry to determine the Treg cell percentage and discovered that the HUWE1 overexpression reduced Treg cell percentage, while this reduction was reversed after the transfection of Lenti-Ets-1 (Figure 6C). Meanwhile, the overexpression of HUWE1 restrained the inhibitory effect of Treg cells on the proliferation of effector T cells, while this trend was reversed after the transfection of Lenti-Ets-1 (Figure 6D). These experimental data authenticated that the HUWE1 overexpression repressed the differentiation and function of Treg cells by decreasing Ets-1.

### 3.7 The HUWE1 Inhibitor has the Function of Relieving ITP in Mice

**FIGURE 7:**
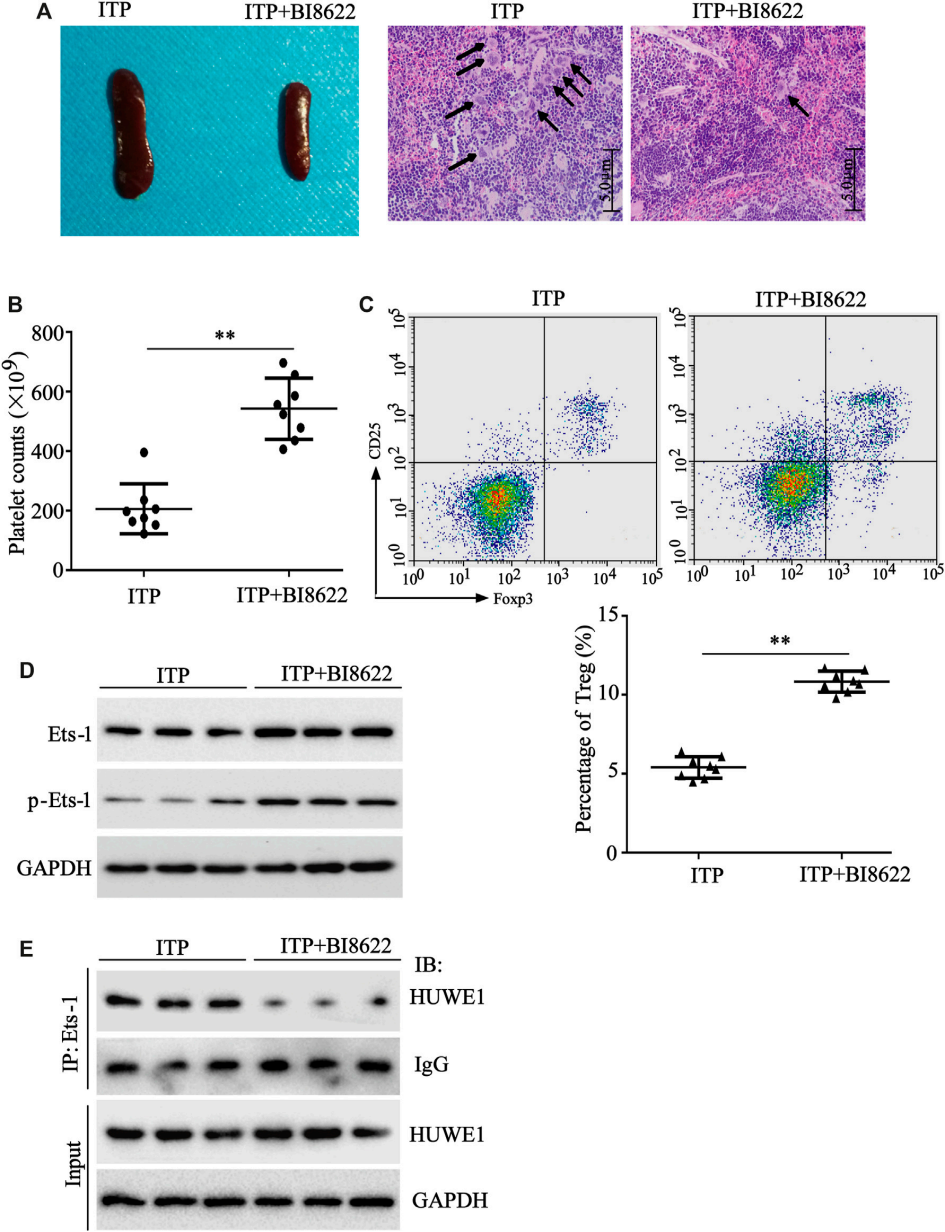
The function of HUWE1 inhibitor on ITP mice. 0.1 mg/kg HUWE1-specific inhibitor BI8622 was intraperitoneally injected into mice during ITP modeling (three times a week). Sixteen male C57BL/6J mice (6–8 weeks old) were divided into the following two groups: ITP group and ITP + BI8622 group, and eight mice were randomly assigned to each group. **(A)** Hematoxylin-eosin (HE) staining was performed to analyze the pathological changes of the spleen in ITP mice (scale bar: 5.0 μm), *n* = 8. **(B)** Analysis of platelet counts in ITP mice (mean ± SEM, *n* = 8). **(C)** Flow cytometry was conducted to analyze the percentage of Treg cells in spleens of ITP mice (mean ± SEM, *n* = 8). **(D)** After the CD4^+^ T cells were isolated from the spleen cells of ITP mice, a Western blot was conducted to detect the protein levels of Ets-1 and p-Ets-1 in cells, *n* = 3. **(E)** The interaction between HUWE1 and Ets-1 in spleen CD4^+^ T cells was confirmed using IP assay. **p < 0.01 vs. ITP.

To further verify the HUWE1 effect on ITP mice *in vivo*, HUWE1-specific inhibitor BI8622 was intraperitoneally injected into mice during ITP modeling. As displayed in Figure 7A, in the BI8622-treated spleen, the lymphocytes were dense, and the red blood sinuses were expanded. In addition, the number of megakaryocytes in the spleen decreased obviously in the BI8622-treated group compared to the model group (black arrows indicated megakaryocytes). Besides, the BI8622 injection elevated the platelet counts in ITP mice (Figure 7B). Flow cytometry results expounded that the BI8622 injection increased the Treg cell percentage in spleens of ITP mice (Figure 7C). Besides, CD4^+^ T cells isolated from spleen cells of ITP mice and Western blot analysis authenticated that the BI8622 injection increased the Ets-1 and p-Ets-1 protein levels (Figure 7D). Meanwhile, the interaction between HUWE1 and Ets-1 in spleen CD4^+^ T cells was assessed and the results corroborated that the interaction between HUWE1 and Ets-1 was prominently reduced after the BI8622 injection, while the HUWE1 expression did not change (Figure 7E). This further confirmed that BI8622 did restrain the interaction between HUWE1 and Ets-1 *in vivo*. Moreover, we confirmed that the BI8622 injection repressed the ubiquitination of Ets-1 by HUWE1, prompting that BI8622 repressed the HUWE1 activity (Supplementary Figure S2). Taken together, the HUWE1 inhibitor had a relieving effect on ITP mice.

## 4 Discussion

Accumulated studies illustrate that CD4^+^ T cell subsets, mainly including Th1, Th2, Th17 and Treg cells, are interrelated to the immune response and ITP pathogenesis (Liu et al., 2013; Zhou et al., 2016). As one of the CD4^+^ T cell subsets, the regulatory function of Treg cells in ITP has gradually attracted wide attention (Zhang et al., 2009; Fu et al., 2016). Similarly, our study expounded that the abnormal number and function of Treg cells led to the immune imbalance of ITP. For the investigation of its mechanism, we authenticated that HUWE1 expression was elevated in the CD4^+^ T cells in peripheral blood from ITP patients and HUWE1 induced the immune imbalance in ITP by reducing the Treg cell number and weakening their immunosuppressive function by targeting the Ets-1 protein degradation. To our knowledge, this study is the first to investigate the role and mechanism of HUWE1 in ITP. Importantly, we further confirmed that the HUWE1 inhibitor had efficacy in relieving ITP mice, providing novel potential candidates for ITP therapeutics.

Ubiquitin modification is one of the post-translational modifications that regulate cell processes through various pathways (Mevissen and Komander, 2017). Ubiquitin modification mainly includes a series of reactions involving ubiquitin-activating enzyme E1, ubiquitin-conjugating enzyme E2 and ubiquitin-ligase E3 (Pao et al., 2018). HUWE1 is a multifaceted HECT domain-containing ubiquitin E3 ligase and functions in various human diseases by identifying different substrates (Gong et al., 2020). Recently, the HUWE1 function in immune-related diseases has attracted widespread attention. For instance, in systemic rheumatic autoimmune diseases, HUWE1 expression is abnormally elevated, implying that HUWE1 might be a biomarker for systemic rheumatic autoimmune diseases (Aqrawi et al., 2019); in immune-induced diseases, HUWE1 regulates the stability and activity of proteins for therapeutic intervention in immune-induced diseases (Singh et al., 2021), prompting that HUWE1 might be one of the candidate molecules for the treatment of the immune-related disease. What similar to the above conclusions, our results also authenticated that HUWE1 expression was elevated in the CD4^+^ T cells in peripheral blood from ITP patients and the mRNA level of HUWE1 was negatively correlated with Treg cell percentage. Our functional findings further corroborated that the interference with HUWE1 elevated the Treg cell number and facilitated its immunosuppressive function, while the HUWE1 overexpression produced the opposite effect, which preliminarily confirmed the pivotal role of HUWE1 in Treg cell number and function in ITP. Besides, Hao et al. demonstrated that the interference with Mule (also named HUWE1) in CD4^+^ T cells results in the restraint of the degradation of the target molecule KLF4, making CD4^+^ T more likely to differentiate into Th17 cells, and preventing differentiation into Treg cells (Hao et al., 2017). This result appears to conflict with our content, but in fact it is not. Our study investigated the abnormal increased HUWE1 expression in CD4^+^ T under ITP pathological conditions, and the dysdifferentiation of CD4^+^ T to Treg by targeting Ets-1. This might be due to the background knockout and the abnormal increase of HUWE1 in the disease state, and the target molecules were different, so the effects on CD4^+^ T differentiation were also different.

Ets-1 is one of the members of the Ets family and the Ets-1 gene is located on chromosome 11 (11q24.3) in humans (Shiu and Jaimes, 2018). Ets-1 is initially confirmed to be an oncogene and recent studies corroborate that Ets-1 also has the function of regulating immune cells (Verschoor and Singh, 2013; Cao et al., 2018). Besides, Ets-1 mediates the development of systemic autoimmune diseases by regulating the immunosuppressive activity of Treg cells (Xiang et al., 2014); in Treg cells, Ets-1 affects Treg cell growth and function by regulating the stable expression of Foxp3, which is conducive to immune homeostasis (Samstein et al., 2012). The above results authenticate that Ets-1 is a key factor regulating the Treg cell growth and function. What intrigues us is that Hou et al. (2016) indicated that dexamethasone ameliorates the function of myeloid-derived suppressor cells by elevating Ets-1, thereby relieving ITP. Thus, restoring the expression and activity of Ets-1 is a new hot spot in the ITP treatment.

Studies authenticate that post-translational modification has a regulatory effect on the Ets-1 expression and that the ubiquitination modification-proteasome pathway is the main pathway leading to the Ets-1 protein degradation (Ji et al., 2007); Nishida et al. (2007) found that the E3 ubiquitin ligase PIASy mediates the proteasome degradation of Ets-1; moreover, the transcriptional activity of Ets-1 is regulated by phosphorylation modifications and the phosphorylation of Thr38 facilitates its transcription (Slupsky et al., 1998; Yang et al., 2019). In this study, we also proved that E3 ubiquitin ligase HUWE1 interacted with Ets-1 and this binding ability was dependent on the phosphorylation level of Ets-1 (Thr38). Our further study demonstrated that HUWE1 facilitated the ubiquitin degradation of Ets-1 protein to restrain the Treg cell differentiation and weaken their immunosuppressive functions.

In conclusion, our experimental data authenticated that E3 ubiquitin ligase HUWE1 reduced the Treg cell number and weakened their immunosuppressive function through the targeted ubiquitin degradation of Ets-1 protein, thus aggravating ITP. The *in vivo* assay confirmed that the HUWE1 inhibitor had the function of alleviating ITP in mice. This study might provide novel insights for ITP treatment in the future.

## Data Availability

The original contributions presented in the study are included in the article/Supplementary Material, further inquiries can be directed to the corresponding author.

## Supplementary Material

Supplementary Figure 1 Influence of HUWE1 on Treg cell immunosuppressive function. CD4^+^ T cells in peripheral blood from ITP patients were transfected with HUWE1 shRNA for 96 h; CD4^+^ T cells in peripheral blood from healthy controls were transfected with Lenti-HUWE1 for 96 h **(A,B)** The CTLA-4 and LAG3 expressions were assessed using qRT-PCR (mean ± SEM, *n* = 3). ***p* < 0.01 vs. shRNA or Lenti. The experiment was repeated three times.

Supplementary Figure 2 Effect of BI8622 on the ubiquitination degradation of Ets-1 by HUWE1. Ubiquitin assay was performed to analyze the ubiquitination of Ets-1 after the BI8622 treatment. The experiment was repeated three times. DMSO: dimethyl sulfoxide.
