# Supplementary figures and images for: HUWE1 Causes an Immune Imbalance in Immune Thrombocytopenic Purpura by Reducing the Number and Function of Treg Cells Through the Ubiquitination Degradation of Ets-1
Source: Front Cell Dev Biol. 2021 Nov 25;9:708562. doi: 10.3389/fcell.2021.708562 (PMC8660631; doi:10.3389/fcell.2021.708562)

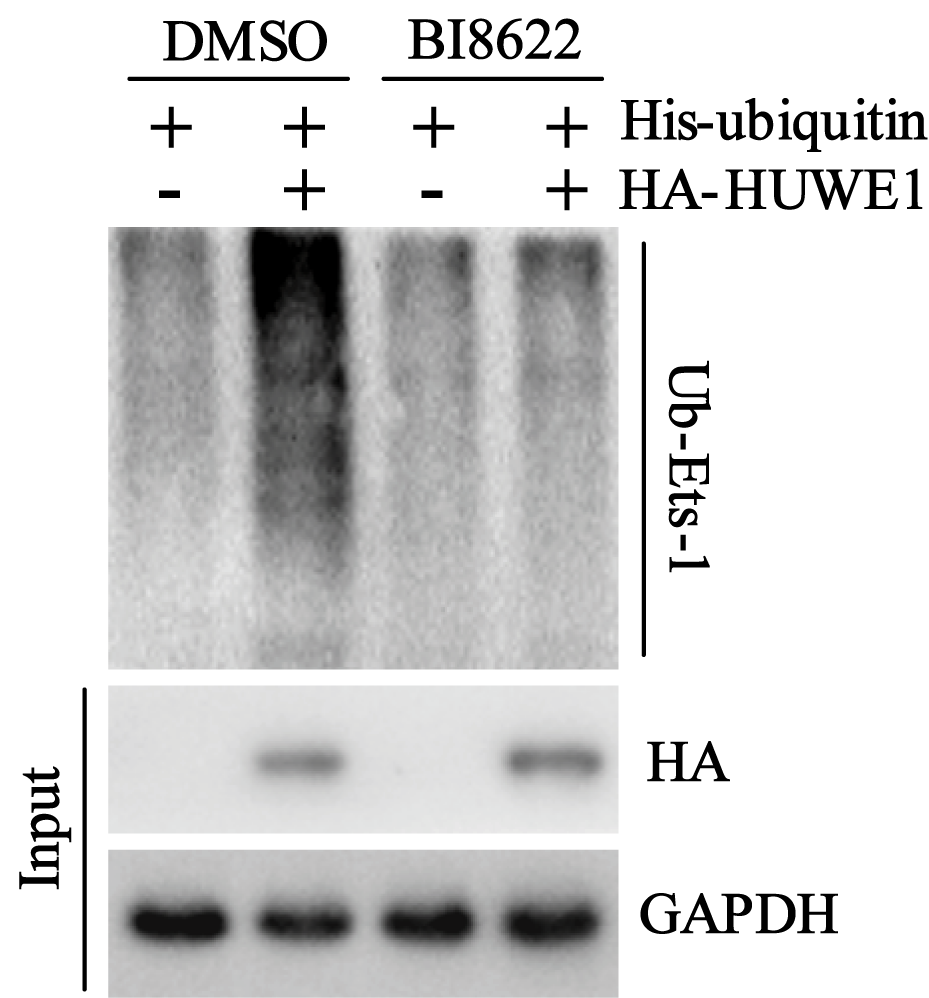

Supplement: Supplementary file 1 [file Image3.TIF]

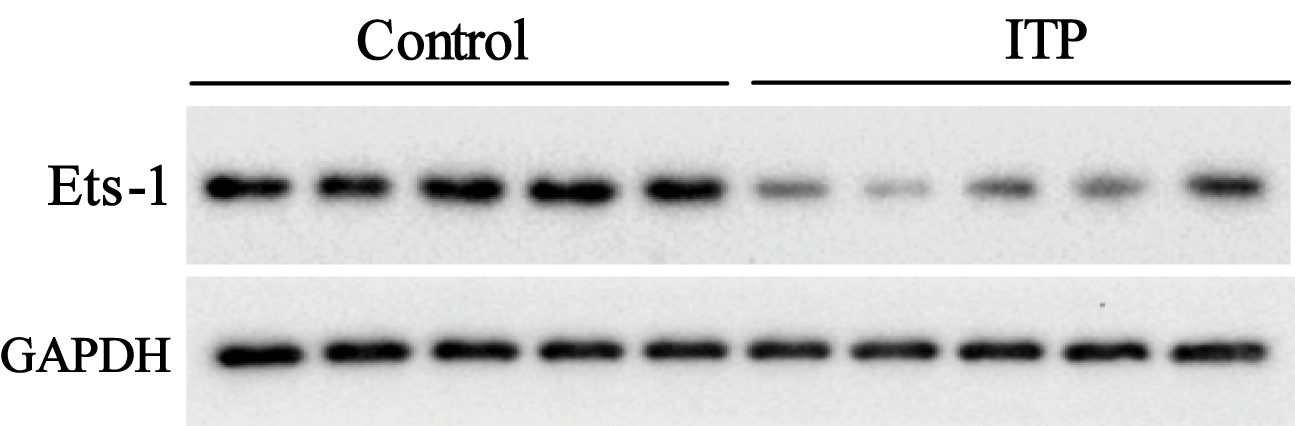

Supplement: Supplementary file 2 [file Image2.TIF]

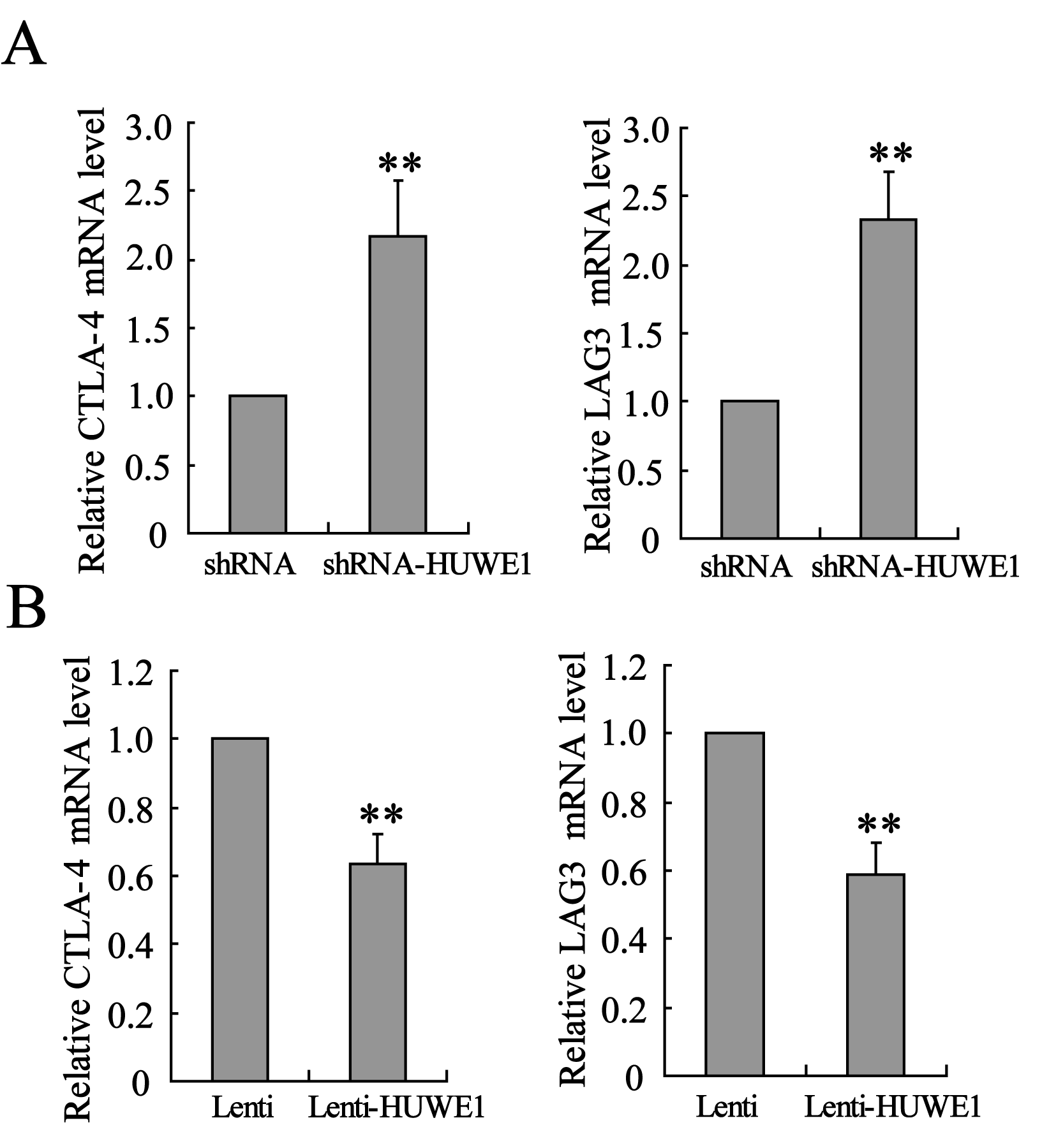

Supplement: Supplementary file 3 [file Image1.TIF]
